# Supplementary material for: Resolving the full spectrum of human genome variation using Linked-Reads
Source: Genome Res. 2019 Apr;29(4):635–45. doi: 10.1101/gr.234443.118 (PMC6442396; doi:10.1101/gr.234443.118)
Supplement: Supplemental Material [file supp_gr.234443.118_Supplemental_longranger-2.2.zip › longranger-2.2.tar.bz2!/lr2.2-public/lib/stan/cmdstan/stan/doc/api/html/agrad_2fwd_2matrix_2multiply_8hpp_source.html]

Stan: src/stan/agrad/fwd/matrix/multiply.hpp Source File


|  |  |
| --- | --- |
|  | Stan  2.5.0  probability, sampling & optimization |


- Main Page
- Related Pages
- Namespaces
- Classes
- Files

- File List
- File Members

All Classes Namespaces Files Functions Variables Typedefs Enumerations Enumerator Friends Macros Pages

- src
- stan
- agrad
- fwd
- matrix

multiply.hpp

Go to the documentation of this file.

1 #ifndef STAN\_\_AGRAD\_\_FWD\_\_MATRIX\_\_MULTIPLY\_HPP

2 #define STAN\_\_AGRAD\_\_FWD\_\_MATRIX\_\_MULTIPLY\_HPP

3

4 #include <vector>

5 #include <boost/math/tools/promotion.hpp>

6 #include <stan/math/matrix/Eigen.hpp>

7 #include <stan/math/matrix/typedefs.hpp>

8 #include <stan/math/error\_handling/matrix/check\_multiplicable.hpp>

9 #include <stan/agrad/fwd/fvar.hpp>

10 #include <stan/agrad/fwd/matrix/typedefs.hpp>

11 #include <stan/agrad/fwd/matrix/to\_fvar.hpp>

12 #include <stan/agrad/fwd/matrix/dot\_product.hpp>

13 #include <stan/agrad/fwd/operators/operator\_multiplication.hpp>

14

15 namespace stan {

16  namespace agrad {

17

18  template<typename T, int R1,int C1>

19  inline

20  Eigen::Matrix<fvar<T>,R1,C1>

21  multiply(const Eigen::Matrix<fvar<T>, R1, C1>& m, const fvar<T>& c) {

22  Eigen::Matrix<fvar<T>,R1,C1> res(m.rows(),m.cols());

23  for(int i = 0; i < m.rows(); i++) {

24  for(int j = 0; j < m.cols(); j++)

25  res(i,j) = c \* m(i,j);

26  }

27  return res;

28  }

29

30  template<typename T,int R2,int C2>

31  inline

32  Eigen::Matrix<fvar<T>, R2, C2>

33  multiply(const Eigen::Matrix<fvar<T>, R2, C2>& m, const double c) {

34  Eigen::Matrix<fvar<T>,R2,C2> res(m.rows(),m.cols());

35  for(int i = 0; i < m.rows(); i++) {

36  for(int j = 0; j < m.cols(); j++)

37  res(i,j) = c \* m(i,j);

38  }

39  return res;

40  }

41

42  template<typename T, int R1,int C1>

43  inline

44  Eigen::Matrix<fvar<T>,R1,C1>

45  multiply(const Eigen::Matrix<double, R1, C1>& m, const fvar<T>& c) {

46  Eigen::Matrix<fvar<T>,R1,C1> res(m.rows(),m.cols());

47  for(int i = 0; i < m.rows(); i++) {

48  for(int j = 0; j < m.cols(); j++)

49  res(i,j) = c \* m(i,j);

50  }

51  return res;

52  }

53

54  template<typename T, int R1,int C1>

55  inline

56  Eigen::Matrix<fvar<T>,R1,C1>

57  multiply(const fvar<T>& c, const Eigen::Matrix<fvar<T>, R1, C1>& m) {

58  return multiply(m, c);

59  }

60

61  template<typename T, int R1,int C1>

62  inline

63  Eigen::Matrix<fvar<T>,R1,C1>

64  multiply(const double c, const Eigen::Matrix<fvar<T>, R1, C1>& m) {

65  return multiply(m, c);

66  }

67

68  template<typename T, int R1,int C1>

69  inline

70  Eigen::Matrix<fvar<T>,R1,C1>

71  multiply(const fvar<T>& c, const Eigen::Matrix<double, R1, C1>& m) {

72  return multiply(m, c);

73  }

74

75  template<typename T, int R1,int C1,int R2,int C2>

76  inline

77  Eigen::Matrix<fvar<T>,R1,C2>

78  multiply(const Eigen::Matrix<fvar<T>,R1,C1>& m1,

79  const Eigen::Matrix<fvar<T>,R2,C2>& m2) {

80  stan::math::check\_multiplicable("multiply(%1%)",m1,"m1",

81  m2,"m2",(double\*)0);

82  Eigen::Matrix<fvar<T>,R1,C2> result(m1.rows(),m2.cols());

83  for (size\_type i = 0; i < m1.rows(); i++) {

84  Eigen::Matrix<fvar<T>,1,C1> crow = m1.row(i);

85  for (size\_type j = 0; j < m2.cols(); j++) {

86  Eigen::Matrix<fvar<T>,R2,1> ccol = m2.col(j);

87  result(i,j) = stan::agrad::dot\_product(crow,ccol);

88  }

89  }

90  return result;

91  }

92

93  template<typename T, int R1,int C1,int R2,int C2>

94  inline

95  Eigen::Matrix<fvar<T>,R1,C2>

96  multiply(const Eigen::Matrix<fvar<T>,R1,C1>& m1,

97  const Eigen::Matrix<double,R2,C2>& m2) {

98  stan::math::check\_multiplicable("multiply(%1%)",m1,"m1",

99  m2,"m2",(double\*)0);

100  Eigen::Matrix<fvar<T>,R1,C2> result(m1.rows(),m2.cols());

101  for (size\_type i = 0; i < m1.rows(); i++) {

102  Eigen::Matrix<fvar<T>,1,C1> crow = m1.row(i);

103  for (size\_type j = 0; j < m2.cols(); j++) {

104  Eigen::Matrix<double,R2,1> ccol = m2.col(j);

105  result(i,j) = stan::agrad::dot\_product(crow,ccol);

106  }

107  }

108  return result;

109  }

110

111  template<typename T, int R1,int C1,int R2,int C2>

112  inline

113  Eigen::Matrix<fvar<T>,R1,C2>

114  multiply(const Eigen::Matrix<double,R1,C1>& m1,

115  const Eigen::Matrix<fvar<T>,R2,C2>& m2) {

116  stan::math::check\_multiplicable("multiply(%1%)",m1,"m1",

117  m2,"m2",(double\*)0);

118  Eigen::Matrix<fvar<T>,R1,C2> result(m1.rows(),m2.cols());

119  for (size\_type i = 0; i < m1.rows(); i++) {

120  Eigen::Matrix<double,1,C1> crow = m1.row(i);

121  for (size\_type j = 0; j < m2.cols(); j++) {

122  Eigen::Matrix<fvar<T>,R2,1> ccol = m2.col(j);

123  result(i,j) = stan::agrad::dot\_product(crow,ccol);

124  }

125  }

126  return result;

127  }

128

129  template <typename T, int C1,int R2>

130  inline

131  fvar<T>

132  multiply(const Eigen::Matrix<fvar<T>, 1, C1>& rv,

133  const Eigen::Matrix<fvar<T>, R2, 1>& v) {

134  if (rv.size() != v.size())

135  throw std::domain\_error("row vector and vector must be same length in multiply");

136  return dot\_product(rv, v);

137  }

138

139  template <typename T, int C1,int R2>

140  inline

141  fvar<T>

142  multiply(const Eigen::Matrix<fvar<T>, 1, C1>& rv,

143  const Eigen::Matrix<double, R2, 1>& v) {

144  if (rv.size() != v.size())

145  throw std::domain\_error("row vector and vector must be same length in multiply");

146  return dot\_product(rv, v);

147  }

148

149  template <typename T, int C1,int R2>

150  inline

151  fvar<T>

152  multiply(const Eigen::Matrix<double, 1, C1>& rv,

153  const Eigen::Matrix<fvar<T>, R2, 1>& v) {

154  if (rv.size() != v.size())

155  throw std::domain\_error("row vector and vector must be same length in multiply");

156  return dot\_product(rv, v);

157  }

158  }

159 }

160 #endif

check\_multiplicable.hpp

typedefs.hpp

stan::agrad::fvar

**Definition:** fvar.hpp:13

typedefs.hpp

operator\_multiplication.hpp

stan::agrad::multiply

Eigen::Matrix< fvar< T >, R1, C1 > multiply(const Eigen::Matrix< fvar< T >, R1, C1 > &m, const fvar< T > &c)

**Definition:** multiply.hpp:21

stan::math::check\_multiplicable

bool check\_multiplicable(const char \*function, const T1 &y1, const char \*name1, const T2 &y2, const char \*name2, T\_result \*result)

**Definition:** check\_multiplicable.hpp:15

dot\_product.hpp

stan::agrad::size\_type

Eigen::Matrix< double, Eigen::Dynamic, Eigen::Dynamic >::Index size\_type

**Definition:** typedefs.hpp:14

stan::agrad::dot\_product

fvar< T > dot\_product(const Eigen::Matrix< fvar< T >, R1, C1 > &v1, const Eigen::Matrix< fvar< T >, R2, C2 > &v2)

**Definition:** dot\_product.hpp:20

fvar.hpp

Eigen.hpp

to\_fvar.hpp


---

[ Stan Home Page ]

*© 2011–2014,
Stan Development Team.*
